# Guanylate binding protein 4 shapes an inflamed tumor microenvironment and identifies immuno-hot tumors

**DOI:** 10.1007/s00432-024-05605-9

**Published:** 2024-02-12

**Authors:** Weijian Zhou, Gaoshaer Yeerkenbieke, Yumei Zhang, Mingwang Zhou, Jin Li

**Affiliations:** 1 Department of Oncology, School of Medicine, Shanghai East Hospital, Tongji University, Shanghai, 200123 China; 2 Department of VIP Clinic, School of Medicine, Shanghai East Hospital, Tongji University, Shanghai, 200123 China

**Keywords:** GBP4, Tumor immunotherapy, Pancancer analysis, Bioinformatics analysis

## Abstract

**Purpose:**

Guanylate binding protein 4 (GBP4) is induced by interferons and various cytokines and has been recognized as functionally relevant in numerous types of human cancers. While the role of GBP4 in cancer has been preliminarily summarized, its correlation with antitumor immunity remains unclear and requires further research.

**Methods:**

First, a comprehensive pan-cancer analysis was conducted, focusing on GBP4’s expression patterns and immunological functions. Subsequently, we explored the correlations between GBP4 and immunological features within the tumor microenvironment (TME) in non-small cell lung cancer (NSCLC) patients. Additionally, we examined the relationships between GBP4 and emerging immunobiomarkers, such as N6-methyladenosine (m6A) genes. Moreover, we assessed the utility of GBP4 in predicting the clinical characteristics and treatment responses of patients with NSCLC.

**Results:**

Pan-cancer analysis revealed that GBP4 plays a positive role in most cancer types via the majority of immunomodulators. Furthermore, GBP4 demonstrated positive associations with immunomodulatory factors, tumor-infiltrating immune cells (TIICs) and inhibitory immune checkpoints. Remarkably, the expression of GBP4 was found to be a predictor of significantly enhanced responsiveness to anti-EGFR therapy and immunotherapy.

**Conclusions:**

GBP4 expression profiles offer a promising avenue for identifying highly immunogenic tumors across a wide spectrum of cancers. GBP4 holds potential as a robust pan-cancer biomarker for assessing the immunological characteristics of tumors, with particular relevance to its ability to predict therapeutic responses, notably in the context of anti-EGFR therapy and immunotherapy.

**Supplementary Information:**

The online version contains supplementary material available at 10.1007/s00432-024-05605-9.

## Introduction

Guanylate binding proteins (GBPs) were first identified in the late 1970s; further studies revealed their mechanism of induction via interferons (IFNs) and other cytokines, and they have been used as markers of IFN responsiveness in both cells and organisms (Vestal 2005). GTPases are highly evolutionarily conserved proteins that offer protection against multiple invading pathogens (Kim et al. 2012). Previous studies have shown that the GBP family is highly important for antibacterial defense and that Guanylate binding protein 4 (GBP4) is a key inflammasome adaptor required for prostaglandin biosynthesis and bacterial clearance by neutrophils (Wandel et al. 2020; Tyrkalska et al. 2016). GBP4 was also found to be highly expressed in multiple cancer types, including melanoma (Gambichler et al. 2022) and colorectal cancer (Xu et al. 2020). In another study, GBP4 expression was shown to be correlated with poor overall survival (OS) in patients with ovarian cancer (Huo et al. 2021). However, the relationship between GBP4 and tumor immunity has not been explored.

Cancers develop within complex tissue environments that consist of diverse types of immune cells. The high heterogeneity within the tumor microenvironment (TME) remains a key obstacle in understanding and treating cancer (Duan et al. 2020). Based on the characteristics of the TME, tumors can be classified into two different types: hot and cold. Hot tumors are infiltrated with more T cells and exhibit greater immune activation, whereas cold tumors exhibit features of T-cell absence or exclusion (Duan et al. 2020). In most cases, patients with hot tumors exhibit increased response rates to immunotherapy (Zemek et al. 2019). Thus, it is essential to use potential biomarkers to identify certain groups of patients who can benefit from immunotherapy by evaluating tumor immunogenicity.

In this study, a pan-cancer analysis of the expression and immunological characteristics of GBP4 was conducted, and the results revealed that GBP4 was strongly correlated with immunological factors in most cancers, especially non-small cell lung cancer (NSCLC). In addition, We found high GBP4 expression could be used to identify an inflamed TME and immuno-hot tumors in NSCLC, and GBP4 could be used to predict the therapeutic efficacy of various therapies in NSCLC patients. Moreover, we further explored the immunological role of GBP4 in multiple cancers. In conclusion, GBP4 is a pan-cancer biomarker that can be used to identify immunogenicity in human cancers.

## Materials and methods

### Data source and preprocessing

The non-small cell lung cancer-normalized gene expression profiles and clinical annotations of The Cancer Genome Atlas (TCGA) datasets were obtained from the online data portal UCSC Xena (https://xenabrowser.net/datapages/). The copy number variant (CNV) information processed by the GISTIC algorithm was also acquired from UCSC Xena. The somatic mutation data were retrieved from the TCGA and then preprocessed with the R package “maftools”. The abbreviations for the mentioned cancer types are listed in Supplementary Table S1.

### Prediction of the immunological characteristics of the TME

Given that the bulk transcriptomic data included both immune and tumor cells from patients, our analysis aimed to define the immunological characteristics within the tumor microenvironment (TME) for each patient. Information on immunomodulators, well-known effector genes of tumor-infiltrating immune cells (TIICs), and specific genes associated with T-cell inflammation and their weighting coefficients was collected from previous studies (Charoentong et al. 2017; Ayers et al. 2017). To assess various attributes of the TME, we employed the ESTIMATE algorithm, which employs gene expression signatures to infer critical parameters such as tumor purity, the ESTIMATE score, the immune score, and the stromal score. In accordance with Yoshihara et al.’s methodology, stromal and immune scores were defined based on gene expression signatures pertaining to stromal tissue and immune cell infiltration. These scores were then amalgamated to generate the ESTIMATE score. Tumor purity, on the other hand, is used to quantify the relative proportion of tumor cells within tumor tissue (Yoshihara et al. 2013). Moreover, to avoid a miscalculation caused by various algorithms when estimating the levels of TIICs, we comprehensively computed the relative abundance of TIICs using the following independent algorithms: TIMER (Li et al. 2020), EPIC (Racle et al. 2017), MCP-counter (Becht et al. 2016), quanTIseq (Finotello et al. 2019), and TISIDB (Ru et al. 2019). Because each step of the cancer immune cycle plays an important role in reflecting the anticancer immune response and determining the fate of tumor cells, we next estimated the activities of each step by performing single-sample gene set enrichment analysis (ssGSEA) according to the expression level of specific signature genes involved in each step (Xu et al. 2018).

### Calculation of the enrichment scores of various gene signatures

We also aimed to determine the oncogenic pathways linked to an inflamed tumor microenvironment (TME), targeted therapy, and response to immunotherapy based on previous research (Hu et al. 2021). To quantify the enrichment scores of these signatures, we employed the R package “GSVA” (Hänzelmann et al. 2013).

### Identification of immune-related m6A genes

According to a recent publication (Shen et al. 2021), we identified 64 m6A regulators and m6A interactive protein-coding genes with a high coexpression tendency and selected genes whose absolute Pearson correlation coefficient with the T-cell-inflamed GEP score was ≥ 0.2 or ≤   – 0.2 and had a *p* value ≤ 0.05.

### Clinical samples

The NSCLC tissue microarrays (TMAs; HLugA150CS04 and HLugS120CS01) were purchased from Outdo Biotech (Shanghai, China). The HLugA150CS04 microarray contains 75 LUAD and 75 adjacent samples. The HLugS120CS01 microarray contains 60 LUSC and 60 adjacent samples. Ethical approval for the study of tissue microarray slides was granted by the Clinical Research Ethics Committee, Outdo Biotech (Shanghai, China).

### Immunohistochemistry

Immunohistochemistry (IHC) was performed directly on the tissue microarray (TMA) following standard protocols. The primary antibodies utilized included anti-GBP4 (diluted 1:2000; Cat. Ab232693; Abcam, Cambridge, UK), anti-PD-L1 (Ready-to-use; Cat. GT2280; GeneTech, Shanghai, China), and anti-CD8 (Ready-to-use; Cat. PA067; Abcarta, Suzhou, China). Antibody staining was visualized using DAB, followed by hematoxylin counterstaining, and the stained sections were subsequently scanned using Aperio Digital Pathology Slide Scanners.

### Semiquantitative scoring

The stained tissue microarray (TMA) was independently assessed by two pathologists. To perform a semiquantitative evaluation of GBP4 and PD-L1 expression exclusively within tumor cells, the percentage of cells exhibiting positive staining was categorized on a scale from 0 to 4, as follows: 0 (< 1%), 1 (1–5%), 2 (6–25%), 3 (26–50%), and 4 (> 50%). The staining intensity was scored on a scale from 0 to 3, as follows: 0 (negative), 1 (weak), 2 (moderate), and 3 (strong). The immunoreactivity score (IRS) was calculated by multiplying the percentage of positive cells by the staining intensity. For the semiquantitative evaluation of CD8 staining, the degree of infiltration was determined by estimating the percentage of cells displaying strong membrane staining intensity among the entire population of stromal cells.

### Prediction of the chemotherapeutic response

We also examined the role of GBP4 in predicting the response to chemotherapy. To identify NSCLC-related drug target genes, we extracted relevant information from the DrugBank database and compared the differences in expression between the high- and low-GBP4 groups. Furthermore, we employed the R package “pRRophetic” to predict individual responses to several commonly used chemotherapeutic drugs. This prediction process was conducted with the Cancer Genome Project (CGP) database (https://www.sciencedirect.com/topics/neuroscience/cancer-genome-project). Within this process, the half-maximal inhibitory concentration (IC50) of each sample was estimated through Ridge regression, and prediction accuracy was assessed via tenfold cross-validation using the CGP training set. All the parameters were set to their default values (Geeleher et al. 2014).

### Statistical methods

All the statistical analyses depicted in the figures were conducted using R version 3.6.0. To assess significant differences in continuous variables between two groups, we employed the Wilcoxon rank-sum test, while categorical variables were compared using the chi-square test. The prognostic significance of each categorical variable was evaluated via the log-rank test. In all analyses, a two-tailed *p* value ≤ 0.05 was regarded as statistically significant unless otherwise specified. Statistical significance was defined as follows: *p value ≤ 0.05, **p value ≤ 0.01, ***p value ≤ 0.001, and ****p value ≤ 0.0001.

## Results

### Expression pattern and immunological role of GBP4 in pancancer

First, a comprehensive analysis was conducted to evaluate the expression and prognostic significance of GBP4 in pan-cancer. Using the TIMER database, we discovered that GBP4 expression was inconsistent in most cancers (Supplementary Fig. S1A). GBP4 mRNA is expressed at a low level in LUAD and LUSC but is expressed at a high level in several other cancer types. Second, a pan-cancer analysis of overall survival (OS) and progression-free survival (PFS) was conducted through the GEPIA database (Supplementary Fig. S1B, C). However, the prognostic value of GBP4 was limited in human cancers. In LGG and PAAD, high expression of GBP4 was related to a worse prognosis, while in OV and SKCM, GBP4 was revealed as a favorable prognostic factor.

**Fig. 1:**
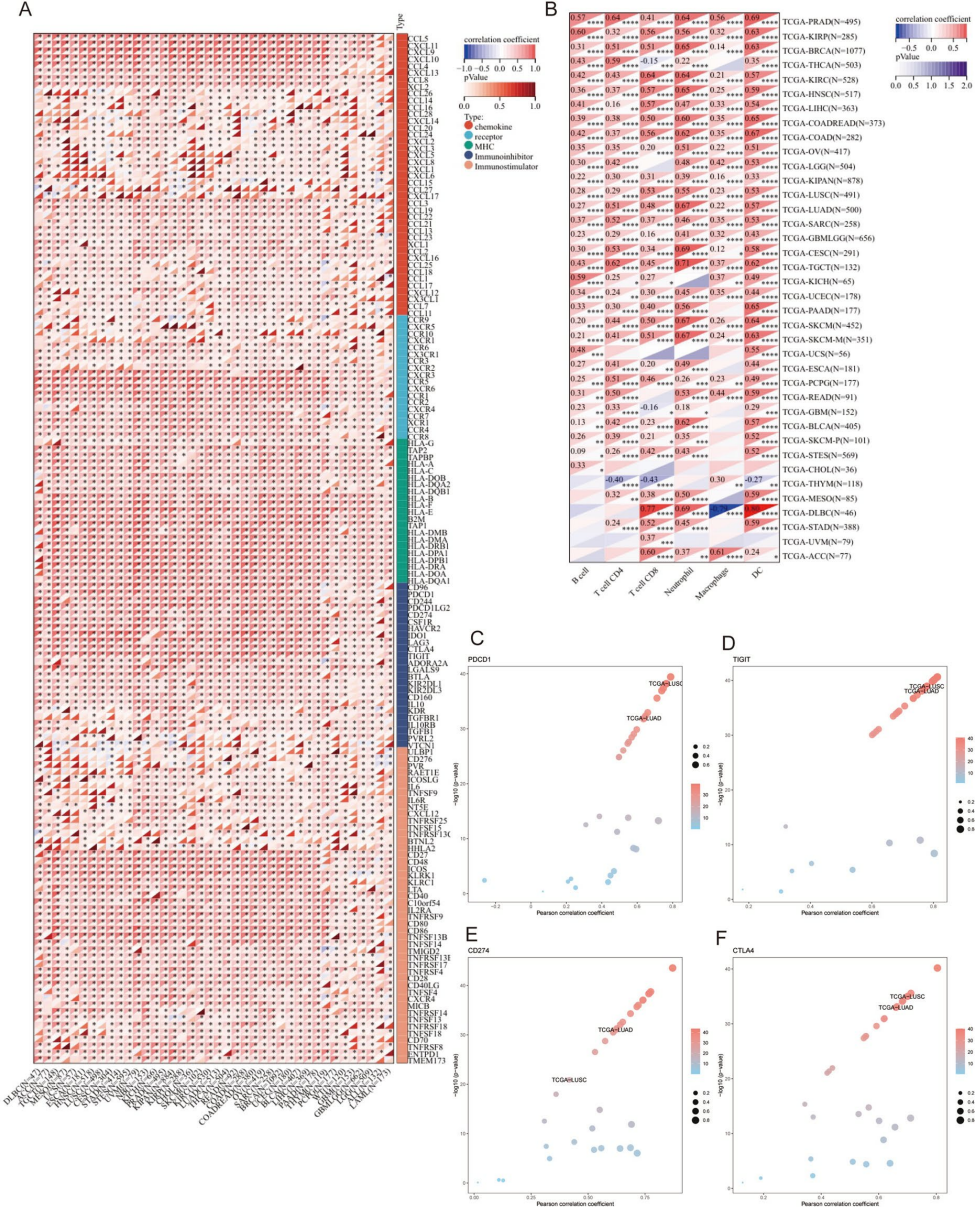
Expression pattern and Immunological role of GBP4 in pan-cancer. **A** Correlations between GBP4 and immunomodulators (chemokines, immunostimulators, MHC, and receptors). The color indicates the correlation coefficient. The asterisks indicate significant differences according to the Pearson analysis. **B** Correlations between GBP4 and TIIC density calculated with the ssGSEA algorithm. The color indicates the correlation coefficient. The asterisks indicate significant differences according to the Pearson analysis. Correlations between GBP4 and 4 immune checkpoint genes: **C** PDCD1, **D** TIGIT, **E** CD274, and **F** CTLA4. The dots represent cancer types. The y-axis represents the Pearson correlation coefficient, while the x-axis represents the × log10 (p value)

Next, a pan-cancer analysis of the immunological features of GBP4 was conducted for all accessible tumor types in the TCGA database. The results revealed that GBP4 plays a positive role with the majority of immunomodulators in most cancer types (Fig. 1A). We next calculated the infiltration levels of tumor-infiltrating immune cells (TIICs) in the TME using the TIMER database. Except for THYM, GBP4 expression was positively correlated with most types of TIICs in multiple cancers (Fig. 1B).

Furthermore, we assessed the correlations between GBP4 and immune checkpoint genes, including TIGIT, CTLA4, CD274 and PDCD1, across cancers. We discovered that GBP4 was positively correlated with these immune checkpoint genes in pan-cancer, and a significant correlation was observed in NSCLC (Fig. 1C–F). Taken together, the above results reveal the potential of GBP4 as an immune-related biomarker in multiple cancers, especially NSCLC.

### GBP4 shapes an inflamed TME in NSCLC

**Fig. 2:**
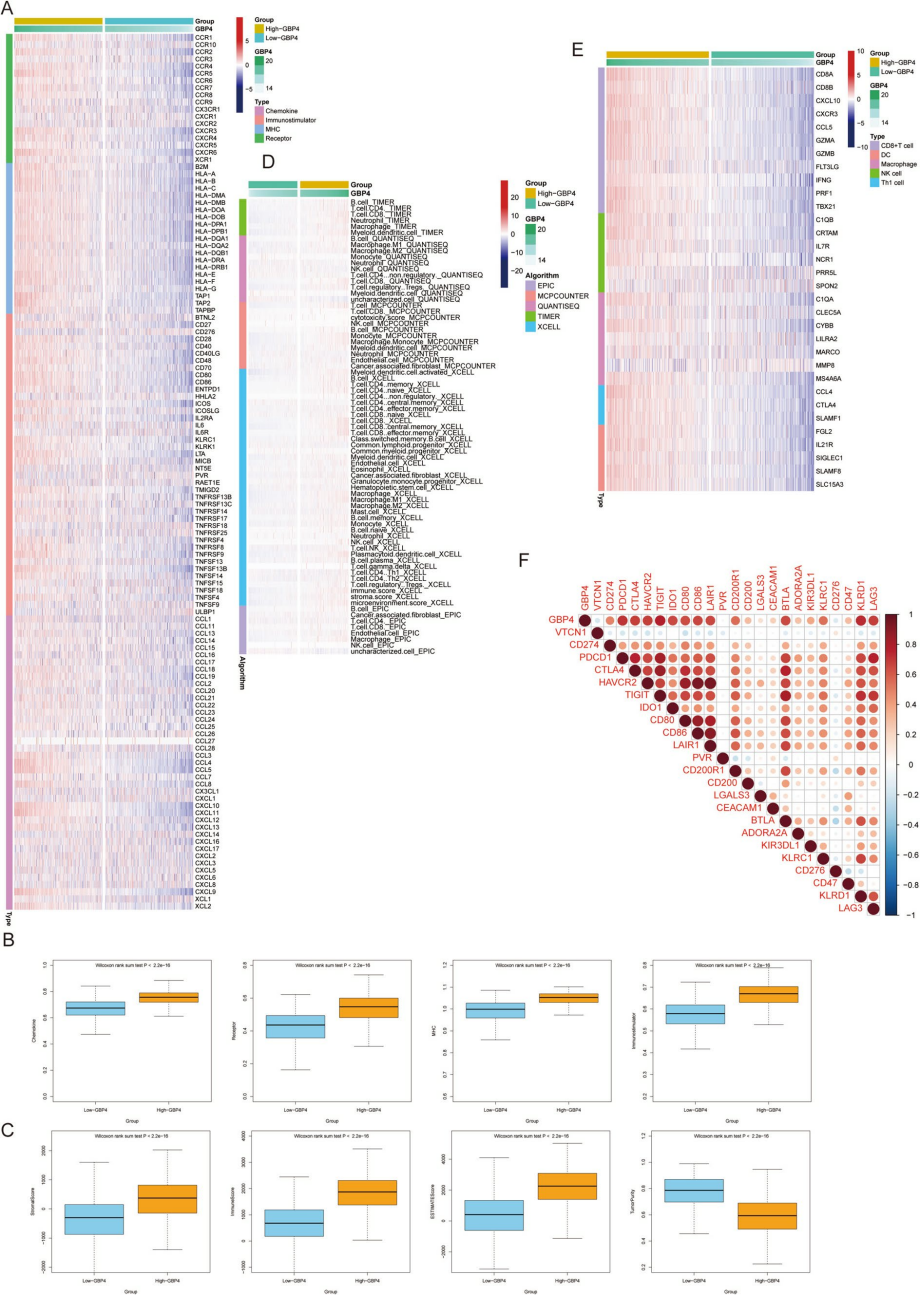
GBP4 shapes an inflamed TME in BLCA. **A**, **B** Expression levels of immunomodulators (chemokines, immunostimulators, MHC, and receptors) in the high- and low-GBP4 groups of NSCLC patients. **C** Distribution of tumor purity, the ESTIMATE score, immune score, and stromal score calculated using the ESTIMATE algorithm in the high- and low-GBP4 groups. **D** The numbers of TIICs calculated using five algorithms (TIMER, EPIC, MCP-counter, quanTIseq, and TISIDB) in the high- and low-GBP4 groups. **E** Expression levels of the gene markers of the common TIICs in the high- and low-GBP4 groups. **F** The activities of the various steps of the cancer immune cycle calculated by the ssGSEA algorithm in the high- and low-GBP4 groups. **G** Correlations between GBP4 and common inhibitory immune checkpoint genes. The color and the values indicate the Pearson correlation coefficient. *p value ≤ 0.05, **p value ≤ 0.01, ***p value ≤ 0.001, and ****p value ≤ 0.0001. NS, not statistically significant

Given the noteworthy correlation observed between GBP4 and immunofactors in NSCLC, we subsequently investigated the immunological role of GBP4 in NSCLC using data from the TCGA database. Our analysis revealed the upregulation of a multitude of chemokines, paired receptors, MHC molecules, and immunomodulators within the high-GBP4 group (Fig. 2A, B). These chemokines and receptors play pivotal roles in attracting effector tumor-infiltrating immune cells (TIICs), including CD8 + T cells, macrophages, and antigen-presenting cells. Furthermore, we employed the ESTIMATE method to evaluate critical TME attributes, including tumor purity, the ESTIMATE score, the immune score, and the stromal score. The high-GBP4 group exhibited higher ESTIMATE scores, immune scores, and stromal scores but lower tumor purity than the low-GBP4 group (Fig. 2C). This observation suggested that tumors displaying high GBP4 expression are accompanied by heightened infiltration of immune cells.

Subsequently, we assessed the level of TIIC infiltration using five independent strategies. Employing diverse algorithms, we consistently noted a substantial upregulation in the infiltration levels of most immune cells within the high-GBP4 group (Fig. 2D). Additionally, we examined the expression of gene markers associated with common immune cells and found that these markers exhibited elevated expression in the high-GBP4 group (Fig. 2E). Inhibitory immune checkpoint genes, such as PD-1/PD-L1, are recognized as highly expressed components within an inflamed TME (Gajewski et al. 2017). As anticipated, GBP4 was strongly correlated with numerous immune checkpoint genes, including CD274, PDCD1, TIGIT, and CTLA4, in NSCLC (Fig. 2F). In summary, GBP4 is strongly associated with an inflamed tumor microenvironment (TME), underscoring its potential diagnostic utility in assessing the immunogenicity of NSCLC.

To understand how GBP4 is associated with immune infiltration in NSCLC, we used TCGA data and found that the expression level of GBP4 was significantly positively correlated with STAT1 both at mRNA level and protein level (Supplementary Fig. S1D, E). STAT1 is the key gene of STAT1/Nk axis (Zemek et al. 2019). This suggests that GBP4 could enhance immune infiltration in NSCLC by participating in STAT1/Nk Axis.

### GBP4 predicts the immune phenotype in NSCLC

**Fig. 3:**
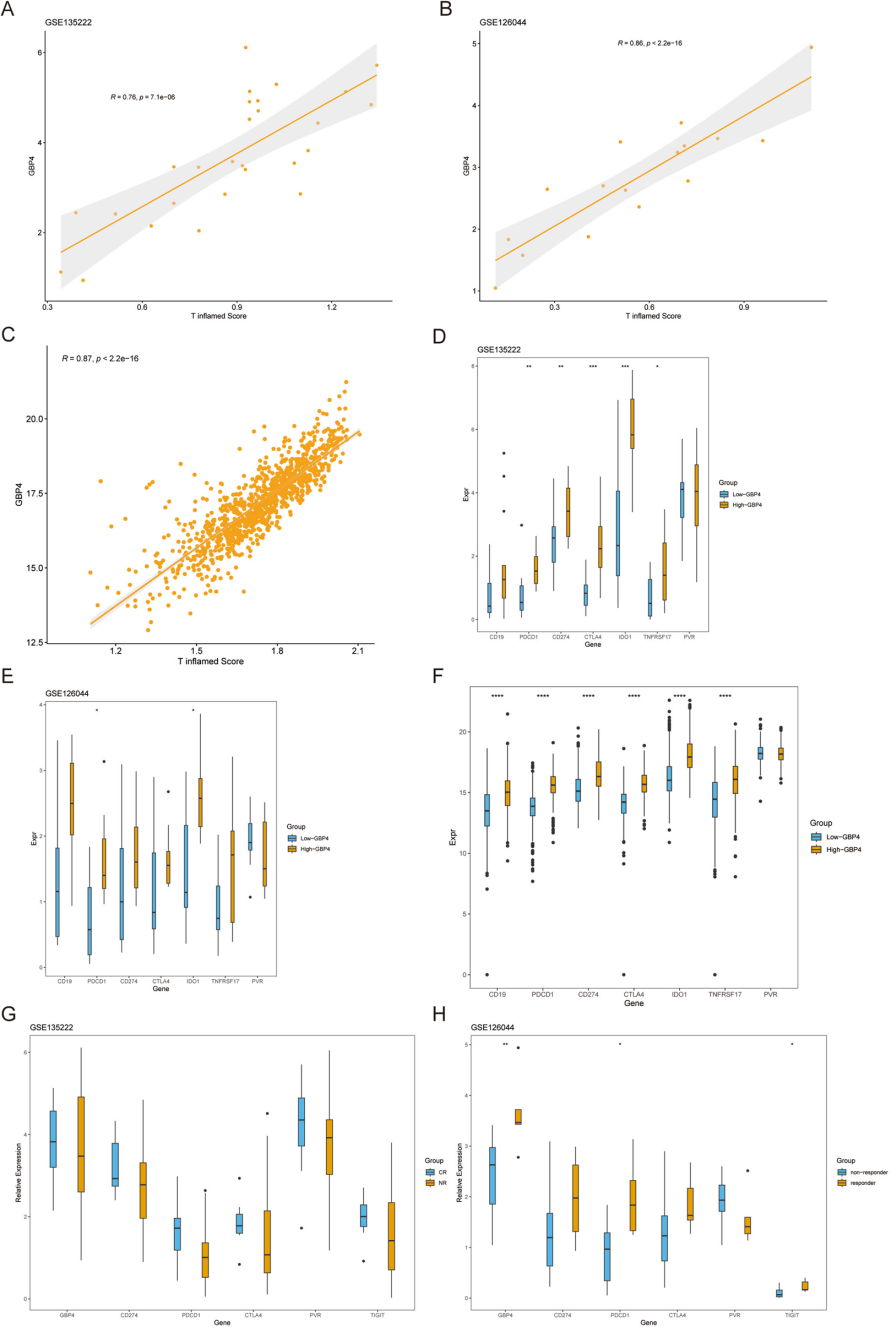
GBP4 can be used to predict the immune phenotype in NSCLC. **A**, **B** T-cell inflamed GEP scores in the high- and low-GBP4 groups in the GSE135222 and GSE126044 datasets. **C** T-cell inflamed GEP scores in the high- and low-GBP4 groups in the TCGA cohort. **D**, **E** Expression levels of immune-related target genes in the high- and low-GBP4 groups in the GSE135222 and GSE126044 datasets. **F** Expression levels of immune-related target genes in the high- and low-GBP4 groups in the TCGA cohort. **G**, **H** Expression levels of GBP4 in the patient cohort receiving PD-L1 treatment

Logically, patients expressing high levels of GBP4 should, in theory, exhibit a more favorable response to immunotherapy owing to’the association of GBP4 with an inflamed tumor microenvironment (TME). The T-cell-inflamed GEP score, established through IFN-γ-related mRNA profiles, serves as a surrogate measure for predicting clinical responses to anti-PD-1 therapy (Ayers et al. 2017). In the context of NSCLC, we observed a positive correlation between GBP4 expression and the T-cell-inflamed GEP score across two datasets from the GEO database, namely, GSE135222 and GSE126044 (Fig. 3A, B). It is widely recognized that the expression levels of immunotargets frequently align with the responsiveness to immunotherapy. Notably, GBP4 expression was positively correlated with the enrichment scores of numerous gene signatures associated with immunotherapy efficacy within the TCGA cohorts (Fig. 3D). Encouragingly, the expression levels of extensively studied immunotargets, such as PDCD1 and IDO1, were markedly elevated in the high-GBP4 group (Fig. 3D–F).

Furthermore, we evaluated the association between GBP4 expression and immunotherapy response in the GSE135222 and GSE126044 datasets. In the GSE135222 dataset, GBP4 expression was significantly higher in the complete response (CR) group than in the nonresponder (NR) group (Fig. 3G). Conversely, in the GSE126044 dataset, GBP4 was notably upregulated in the responder group compared with the nonresponder group (Fig. 3H). This finding implies that the response to immunotherapy likely depends on multiple factors, and the expression analysis of a single gene may be useful for discerning efficacy. Nevertheless, it is worth highlighting that GBP4 is associated with immune checkpoint genes and immune phenotypes, at least within the context of NSCLC.

### GBP4 correlates with emerging immunobiomarkers in NSCLC

**Fig. 4:**
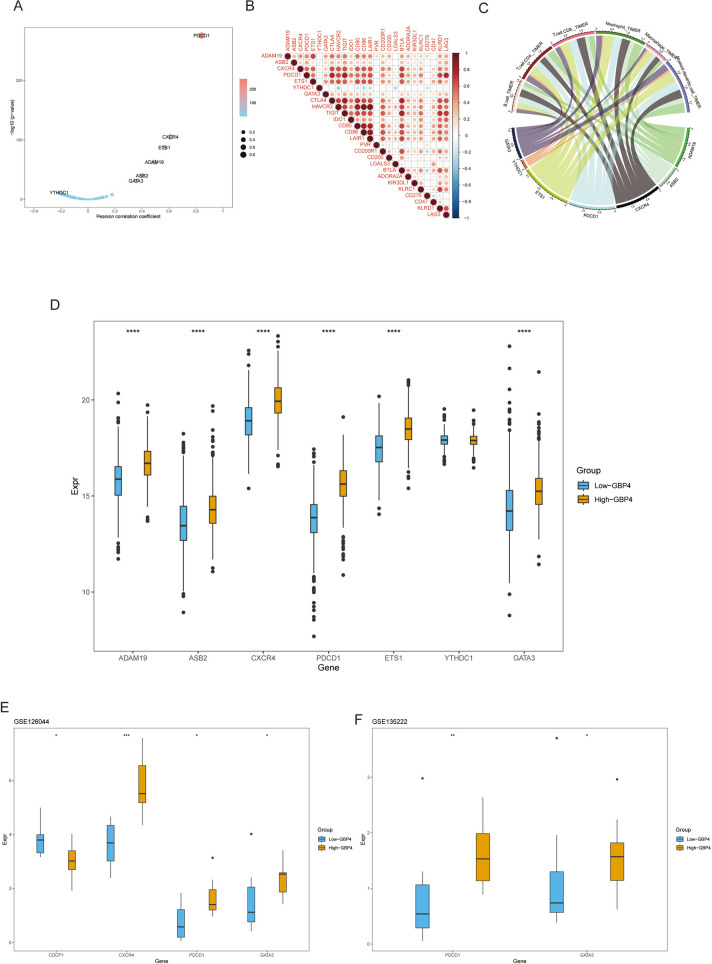
Correlations between GBP4 and m6A gene expression in NSCLC. **A** Correlation between the T-cell inflamed GEP score and m6A gene expression. The dots represent different m6A genes. The y-axis represents the Pearson correlation coefficient, while the x-axis represents the − log10 (p value). **B** Correlations between immune-related m6A genes and immune checkpoint gene expression. **C** Correlations between immune-related m6A gene expression and TIIC levels estimated by TIMER. **D**–**F** Expression levels of immune-related m6A genes in the high- and low-GBP4 groups in the TCGA cohort. *p value ≤ 0.05, **p value ≤ 0.01, ***p value ≤ 0.001, and ****p value ≤ 0.0001. NS: not statistically significant

N6-methyladenosine (m6A) is a modification occurring at the N6 position of adenosine and plays pivotal roles in the initiation and progression of diverse cancer types (Zhang et al. 2020a; Chen et al. 2020). Additionally, accumulating evidence underscores the association of m6A regulators with antitumor immunity, positioning them as potential indicators for predicting tumor immunogenicity (He et al. 2021; Zhang et al. 2020b). In accordance with the findings of a recent publication (Shen et al. 2021), we identified 64 m6A regulators and m6A interactive protein-coding genes that exhibited substantial coexpression tendencies. Of these genes, 63 were expressed in NSCLC, and among these genes, 7 were correlated with the T-cell-inflamed GEP score (Supplementary Table S2), underscoring the close interconnection between m6A networks and antitumor immunity. For further analysis, we selectively extracted genes that met the following criteria: Pearson correlation coefficient ≥ 0.2 or ≤   −  0.2 (Fig. 4A). These six m6A-related genes were positively correlated with the T-cell-inflamed GEP score and were also found to be positively associated with the expression of immune checkpoint genes and the infiltration of immune cells, while the remaining genes were negatively correlated with immune checkpoint gene expression and immune cell infiltration (Fig. 4B, C). Subsequently, we explored the relationship between GBP4 and these m6A genes. Within the high-GBP4 group, the expression of the six m6A-related genes that were positively correlated with the T-cell-inflamed GEP score was significantly upregulated, while the remaining genes showed no significant difference in expression in the TCGA cohort (Fig. 4D). Furthermore, most of these findings were validated within the GEO datasets GSE135222 and GSE126044 (Fig. 4E, F). Additionally, the mutation rates of immune-related m6A genes were variable between the high- and low-GBP4 groups.

In summary, m6A-related genes exhibited noteworthy associations with antitumor immunity and demonstrated correlations with GBP4 (Fig. 4).

### GBP4 is associated with immune and clinical phenotypes in the TMA cohort

**Fig. 5:**
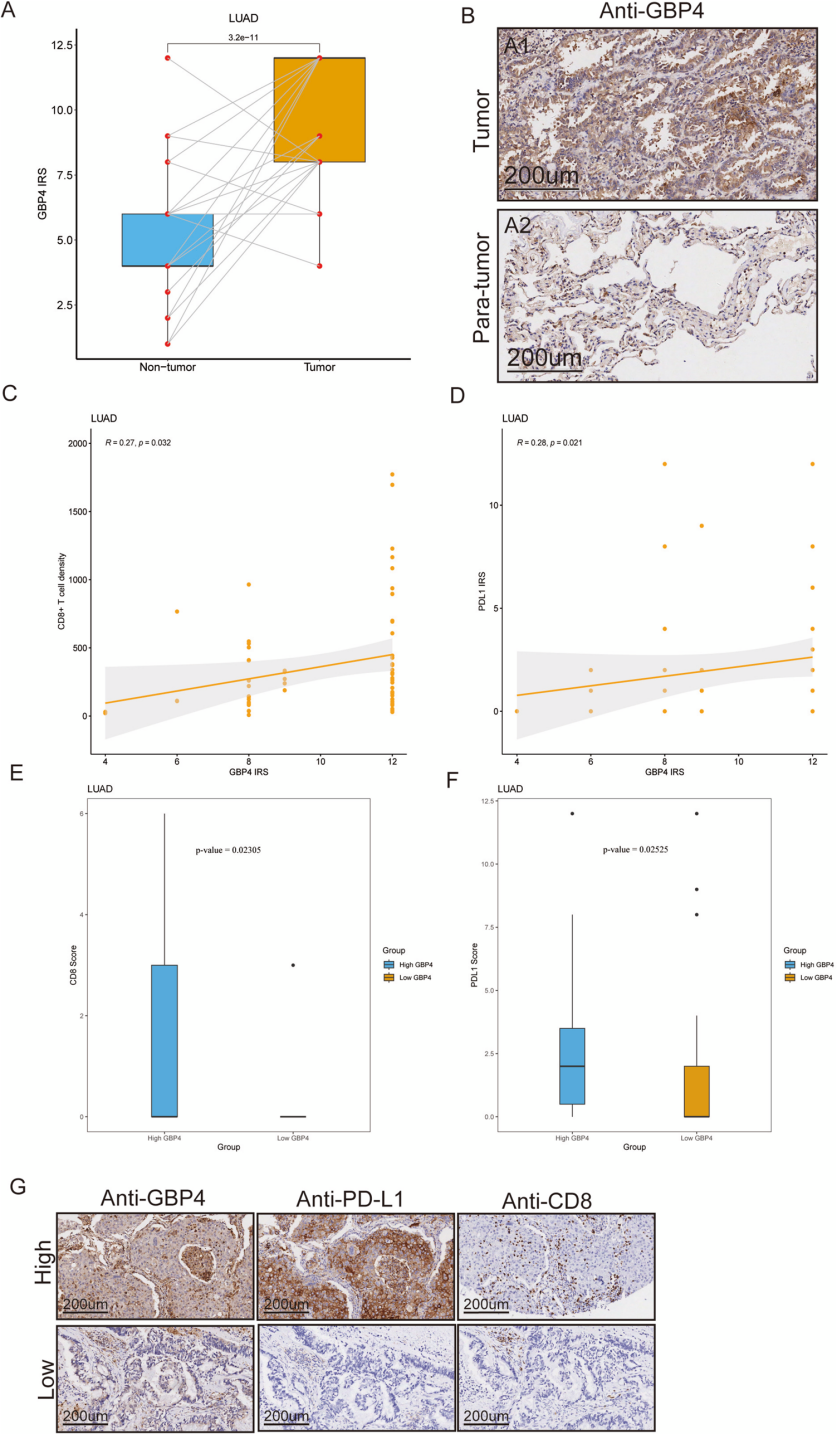
The role of GBP4 in predicting clinical and immune phenotypes in the TMA. **A** Expression levels of GBP4 in tumor and paratumor tissues. **B** Representative images revealing GBP4 expression in tumor and paratumor tissues using anti-GBP4 staining. Magnification, 200 × . **C** Correlation between GBP4 and PD-L1 expression. **D** Correlation between GBP4 expression and CD8 + T-cell infiltration levels. **E** CD8 + T-cell infiltration levels in the high- and low-GBP4 groups. **F** Expression level of PD-L1 in the high- and low-GBP4 groups. **G** Representative images revealing CD8 + T-cell infiltration and PD-L1 expression in the high- and low-GBP4 groups. Magnification, 200 ×

To confirm the above results, we also used two TMA cohorts that included 135 NSCLC and paracancerous tissue samples for validation (Supplementary Fig. S2A–H). GBP4 expression was more highly upregulated in tumor tissues than in paracancerous tissues at the protein level (Fig. 5A, B; Supplementary Fig. S3A, B). Moreover, GBP4 was positively correlated with CD8 + T-cell infiltration and PD-L1 expression in the LUAD cohort (Fig. 5C, D). In addition, the LUAD cohort was classified into low- and high-expression groups based on the median level of GBP4 expression, and we found that the infiltration level of CD8 + T cells and expression of PD-L1 were higher in the high-GBP4 group (Fig. 5E–G). In the LUSC cohort, GBP4 was positively correlated with PD-L1 expression (Supplementary Fig. S3C) but not significantly correlated with CD8 + T-cell infiltration. Overall, GBP4 expression is correlated with immune phenotypes and clinical features in NSCLC.

### GBP4 Can be used to predict therapeutic opportunities in NSCLC patients with immunological effects

**Fig. 6:**
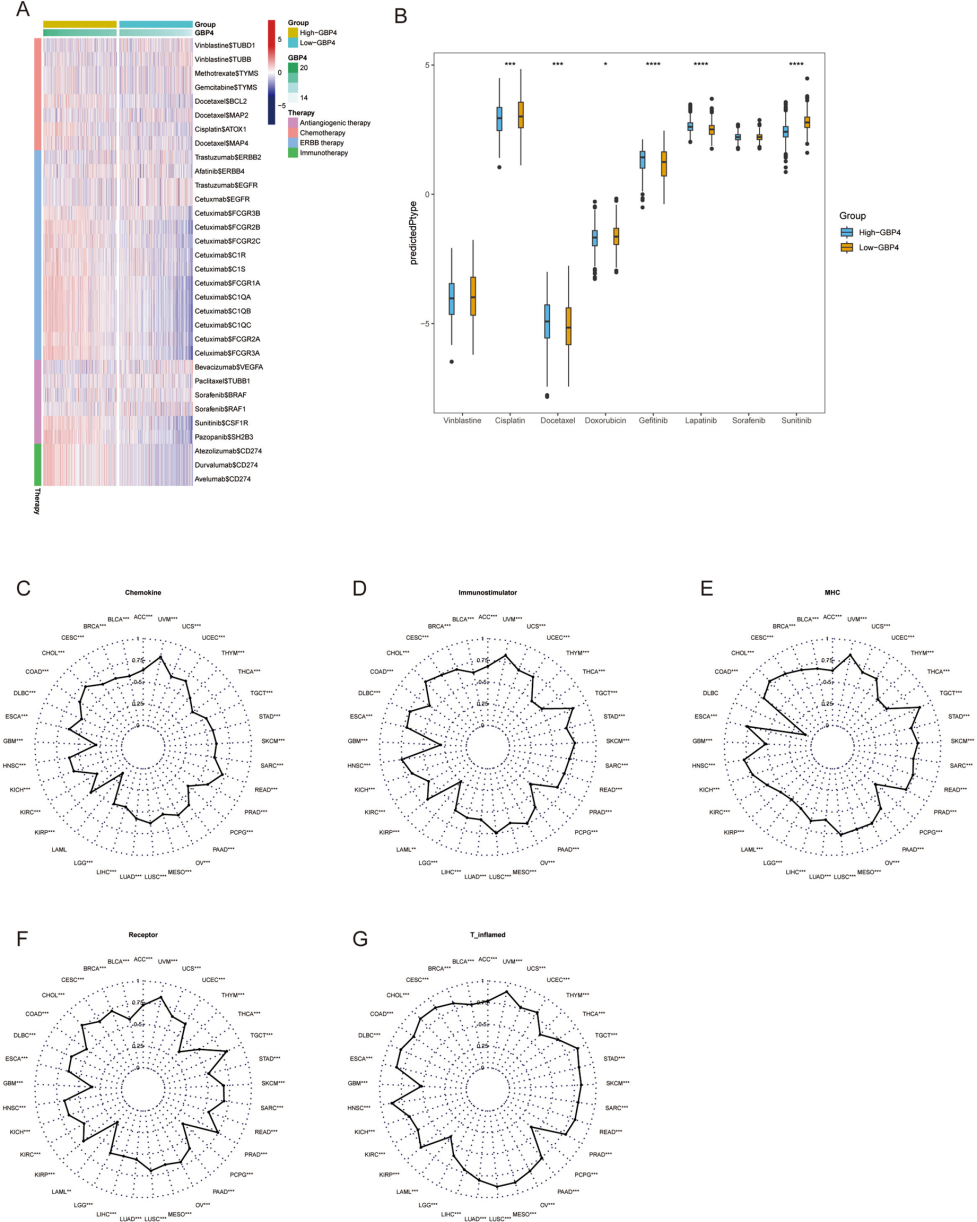
GBP4 Can be used to predict therapeutic opportunities and immunological effects in NSCLC and pan-cancer. **A** Correlations between GBP4 and drug target genes obtained from the DrugBank database. **B** Differences in the IC50s of common anticancer drugs calculated using the “pRRophetic” R package between the high- and low-GBP4 groups. **C** Pan-cancer correlations between GBP4 and the chemokine score. **D** Pan-cancer correlations between GBP4 and the immunostimulatory score. **E** Pan-cancer correlations between GBP4 and the MHC score. **F** Pan-cancer correlations between GBP4 and the receptor score. **G** Pan-cancer correlations between GBP4 and T-cell inflamed GEP score

We evaluated GBP4 expression and its correlation with the response to other therapies. The results obtained from our analysis of the DrugBank database (https://go.drugbank.com/) revealed a notably higher response rate to anti-EGFR therapy and immunotherapy in the high-GBP4 group (Fig. 6A). Moreover, the IC50s of the anticancer drugs were estimated according to the pRRophetic algorithm. The results showed that patients with high GBP4 expression tend to be sensitive to certain therapeutic options (Fig. 6B). We used GEO datasets GSE135222 (Supplementary Fig. S4A) and GSE126044 (Supplementary Fig. S4B) to verify the result. We found that patients with high GBP4 expression were more sensitive to sunitinib. Subsequently, we used lung adenocarcinoma cell line A549 and lung squamous cell carcinoma cell line NCI-H520 for verification.And result is consistent (Supplementary Fig. S4C–F).

All these data illustrated that GBP4 was associated with an inflamed TME and could be used to identify immuno-hot tumors in NSCLC. However, as previously shown in Fig. 1, with the exception of some tumor types, GBP4 may be a pan-cancer indicator of tumor immunogenicity. We analyzed the correlations between GBP4 and chemokine, MHC, immunostimulator, and receptor scores. Except for LAML, GBP4 was positively correlated with the chemokine score (Fig. 6C); for all cancer types assessed in the TCGA, GBP4 was positively correlated with the immunostimulator score (Fig. 6D); except for DLBC, GBP4 was positively correlated with the MHC score (Fig. 6E); for all cancer types assessed in the TCGA, GBP4 was positively correlated with both the receptor score and T-cell-inflamed GEP score (Fig. 6F, G). Taken together, these data suggest that GBP4 is a pan-cancer marker for high immunogenicity, except in a few tumor types.

## Discussion

Non-small cell lung cancer (NSCLC) ranks among the malignancies associated with the highest morbidity and mortality rates. Approximately 70% of NSCLC patients receive diagnoses at advanced stages, with a 5-year survival rate of approximately 23% (Miller et al. 2019). Currently, several immunotherapeutic agents are expected to be more widely used in the clinic, among which anti-CTLA-4, anti-PD-1, and anti-PD-L1 therapies are the most prominent. Ipilimumab, which targets CTLA-4, has shown good efficacy in combination with radiotherapy or chemotherapy regimens (Wilkins et al. 2019; Lynch et al. 2012). However, it is important to acknowledge that not all patients respond favorably to immunotherapy. As research progresses, it becomes increasingly evident that the heterogeneity and dynamic changes within the tumor immune microenvironment (TIME) have a substantial influence on the response to immunotherapy.

The tumor microenvironment (TME) is a dynamic system primarily comprising immune cells, stromal cells, tumor cells, and a complex milieu of cytokines and chemokines. Among these components, immune and stromal cells are the two principal nontumor constituents of the TME and have significant diagnostic and prognostic influence in cancer (Jia et al. 2018). Hence, the exploration of immune-related factors within the TME and the identification of potential biomarkers associated with TIME characteristics are highly important (Liu and Sun 2021). This endeavor is essential for the effective identification of individuals who may benefit from immunotherapy in clinical practice.

PD-L1 is a common and effective clinical predictor that can guide the use of immune checkpoint inhibitors (ICIs) for the treatment of non-small cell lung cancer (NSCLC) due to its robust performance as an immunotherapy biomarker (Hurkmans et al. 2020). However, relying solely on PD-L1 as a screening indicator for identifying patients suitable for ICI treatment reveals certain limitations within clinical data. Previous research has indicated that IFN-γ plays an important role in significantly augmenting PD-L1 expression. Moreover, PD-L1 is highly expressed in the context of immune “hot” tumors and is significantly correlated with TIME characteristics (Qian et al. 2018).

In our research, we discovered that GBP4 was upregulated in tumors from patients responsive to immunotherapy and associated with immunomodulators in the TIME utilizing a panel of public cohorts. In addition, we also conducted a pan-cancer analysis and found that GBP4 was positively correlated with the inflamed TIME, and the positive correlation between GBP4 and PD-L1 was validated in multiple in-house cohorts. These evidence enhanced the correlation of GBP4 with anti-tumor immunity. IFN-γ/STAT1 signaling is essential for both GBP4 and PD-L1 expression, which explains the co-expression pattern of GBP4 with PD-L1 and the elevation of GBP4 and PD-L1 in the inflamed tumors. However, further studies on GBP4 in tumors are lacking; thus, the functional role of GBP4 is still unclear.

In essence, this investigation sheds light on the immunological significance of GBP4 and underscores its potential as a predictive biomarker for assessing immunotherapy responses within NSCLC datasets. However, in the TMA, GBP4 did not show the same correlation in LUSC as it did in LUAD. Consequently, further validation involving a larger patient population receiving immunotherapy with immune checkpoint inhibitors (ICIs) is warranted.

## Conclusions

The current study revealed that GBP4 expression shapes an inflamed TME in NSCLC and can be used to predict the immune and clinical characteristics of patients with NSCLC. Moreover, the results of the pan-cancer analysis suggest that GBP4 can be used to predict high immunogenicity in most cancers. Overall, GBP4 might be a promising biomarker for identifying tumor immunogenicity and guiding immunotherapy.

## Supplementary Information

Below is the link to the electronic supplementary material.

Supplementary file1 (DOCX 8 KB)

Supplementary file2 (DOCX 16 KB)

Supplementary file3 (TIF 6065 KB) Supplementary Figure 1.Pan-cancer analysis of expression and prognostic value of GBP4. (A)Expression of IFITM3 across different cancer types. Heatmap of prognostic value of GBP4 in predicting (B) OS and (C) PFS in pan-cancer analysis. Expression of STAT1 is positively correlated with GBP4 at mRNA level (D) and protein level (E)

Supplementary file4 (TIF 8718 KB) Supplementary Figure 2. The landscape of TMA HLugS120CS01 and HLugA150CS04. (A) Distribution of samples in TMA HLugS120CS01. Blue dots: tumor samples; Green dots: para-tumor samples. (B) The landscape of anti-GBP4 staining TMA HLugS120CS01. (C) The landscape of anti-PD-L1 staining TMA HLugS120CS01. (D) The landscape of anti-CD8 staining TMA HLugS120CS01. (E) Distribution of samples in TMA HLugA150CS04. Blue dots: tumor samples; Green dots: para-tumor samples. (F) The landscape of anti-GBP4 staining TMA HLugA150CS04. (G) The landscape of anti-PD-L1 staining TMA HLugA150CS04. (H) The landscape of anti-CD8 staining TMA HLugA150CS04

Supplementary file5 (TIF 5324 KB) Supplementary Figure 3. The role of GBP4 in predicting clinical and immune phenotypes in the recruited TMA cohort. (A) Expression levels of GBP4 in tumor and paratumor tissues. (B) Representative images revealing GBP4 expression in tumor and paratumor tissues using anti-GBP4 staining. Magnification, 200X

Supplementary file6 (TIF 26989 KB) Supplementary Figure 4. GBP4 Can be used to predict therapeutic opportunities in NSCLC. Differences in the IC50s of common anticancer drugs calculated using the GSE datasets GSE135222 (A) and GSE126044 (B). (C,D) Differences in the IC50s of sunitinib in A549 cell line. (E,F) Differences in the IC50s of sunitinib in NCI-H520 cell line

## Data Availability

The datasets generated and analyzed during the current study are available from the corresponding author on reasonable request.
