# Supplementary material for: Guanylate binding protein 4 shapes an inflamed tumor microenvironment and identifies immuno-hot tumors
Source: J Cancer Res Clin Oncol. 2024 Feb 12;150(2):90. doi: 10.1007/s00432-024-05605-9 (PMC10861698; doi:10.1007/s00432-024-05605-9)
Supplement: Supplementary file 1 — Supplementary file1 (DOCX 8 KB) [file 432_2024_5605_MOESM1_ESM.docx]

Table S1. Table of abbreviations. Abbreviation Full name

ACC Adrenocortical carcinoma BLCA Bladder urothelial carcinoma BRCA Breast invasive carcinoma

CESC Cervical squamous cell carcinoma and endocervical adenocarcinoma CHOL Cholangio carcinoma

COAD Colon adenocarcinoma

DLBC Lymphoid neoplasm diffuse large B-cell lymphoma ESCA Esophageal carcinoma

GBM Glioblastoma multiforme

HNSC Head and neck squamous cell carcinoma KICH Kidney chromophobe carcinoma

KIRC Kidney renal clear cell carcinoma KIRP Kidney renal papillary cell carcinoma LAML Acute myeloid leukemia

LGG Brain lower grade glioma

LIHC Liver hepatocellular carcinoma

LUAD Lung adenocarcinoma

LUSC Lung squamous cell carcinoma MESO Mesothelioma

OV Ovarian serous cystadenocarcinoma

PAAD Pancreatic adenocarcinoma

PCPG Pheochromocytoma and paraganglioma PRAD Prostate adenocarcinoma

READ Rectum adenocarcinoma

SARC Sarcoma

SKCM Skin cutaneous melanoma

STAD Stomach adenocarcinoma

TGCT Testicular germ cell tumors

THCA Thyroid carcinoma

THYM Thymoma

UCEC Uterine corpus endometrial carcinoma UCS Uterine carcinosarcoma

UVM Uveal melanoma
