# Supplementary material for: Guanylate binding protein 4 shapes an inflamed tumor microenvironment and identifies immuno-hot tumors
Source: J Cancer Res Clin Oncol. 2024 Feb 12;150(2):90. doi: 10.1007/s00432-024-05605-9 (PMC10861698; doi:10.1007/s00432-024-05605-9)
Supplement: Supplementary file 2 — Supplementary file2 (DOCX 16 KB) [file 432_2024_5605_MOESM2_ESM.docx]

Table S3. Correlation between T cell inflamed score and m6A genes.

| m6A genes | coefficient | pvalue |
| --- | --- | --- |
| PDCD1 | 0.841899201 | 2.5688E-276 |
| CXCR4 | 0.610578107 | 9.5862E-106 |
| ETS1 | 0.565842451 | 8.19526E-88 |
| ADAM19 | 0.491201787 | 2.32409E-63 |
| ASB2 | 0.399724388 | 1.31847E-40 |
| GATA3 | 0.355331026 | 7.25017E-32 |
| YTHDC1 | -0.213594561 | 4.84954E-12 |
| HNRNPA2B1 | -0.193375295 | 4.30664E-10 |
| SOX2 | -0.189332282 | 9.99224E-10 |
| SRSF11 | -0.188722484 | 1.13267E-09 |
| SRSF6 | -0.183502839 | 3.25659E-09 |
| METTL3 | -0.18257515 | 3.91657E-09 |
| BCL2 | 0.177634863 | 1.02981E-08 |
| USP7 | -0.169037375 | 5.19635E-08 |
| YTHDF2 | -0.152671341 | 9.07433E-07 |
| METTL16 | -0.152156555 | 9.88247E-07 |
| RBM15B | -0.14091141 | 5.94111E-06 |
| PTEN | 0.138355275 | 8.7679E-06 |
| YTHDF1 | -0.129074837 | 3.40232E-05 |
| BRD4 | -0.127070639 | 4.50686E-05 |
| ALKBH5 | -0.116192439 | 0.000192953 |
| HIF1A | 0.113104468 | 0.00028525 |
| WTAP | 0.111441373 | 0.000350695 |
| ZMYM1 | -0.110467874 | 0.000395256 |
| LEF1 | 0.108908781 | 0.000477766 |
| SRF | -0.108072844 | 0.000528355 |
| TAZ | -0.106589989 | 0.000630547 |
| EIF3A | -0.099020642 | 0.001502797 |
| RELA | 0.099020081 | 0.001502891 |
| MZF1 | -0.096110592 | 0.002067094 |
| ZC3H13 | -0.090514104 | 0.003728242 |
| SEC62 | -0.089975087 | 0.003939794 |
| TP53 | 0.084142005 | 0.00703142 |
| CDCP1 | 0.076239722 | 0.01462891 |
| SP1 | -0.067984539 | 0.029523137 |
| IGF2BP2 | -0.06362927 | 0.041679212 |
| CTNNB1 | 0.062120515 | 0.04677624 |
| RBMX | -0.06143749 | 0.049250446 |
| TK1 | 0.060492564 | 0.052853357 |
| KIAA1429 | -0.05238049 | 0.093718929 |
| SRSF3 | -0.051096297 | 0.102059187 |
| ITGA6 | -0.050462705 | 0.106386019 |
| UBE2C | 0.048160734 | 0.123339034 |
| SOCS2 | 0.046335367 | 0.138222806 |
| IGF2BP1 | -0.042143181 | 0.177596395 |
| AFF4 | -0.040076302 | 0.199836627 |
| IKBKB | -0.037725504 | 0.227524091 |
| NANOG | -0.029139217 | 0.351352193 |
| MYB | 0.027098145 | 0.386125874 |
| YTHDC2 | 0.02525597 | 0.419247319 |
| IGF2BP3 | 0.023278654 | 0.456590939 |
| HDGF | -0.017568904 | 0.574227495 |
| NOTCH1 | -0.017083026 | 0.584862342 |
| E2F1 | -0.010942858 | 0.726394704 |
| FOXM1 | -0.009882491 | 0.751990567 |
| SP2 | 0.008553368 | 0.784459469 |
| HNRNPC | -0.008377965 | 0.788773845 |
| METTL14 | 0.007228476 | 0.817202327 |
| KEAP1 | -0.006740262 | 0.829351938 |
| YTHDF3 | -0.006182482 | 0.843282627 |
| PHLPP2 | -0.004141209 | 0.894650376 |
| FSCN1 | -0.001895821 | 0.951660352 |
| FTO | 0.000517866 | 0.986787985 |
