# Supplementary figures and images for: Guanylate binding protein 4 shapes an inflamed tumor microenvironment and identifies immuno-hot tumors
Source: J Cancer Res Clin Oncol. 2024 Feb 12;150(2):90. doi: 10.1007/s00432-024-05605-9 (PMC10861698; doi:10.1007/s00432-024-05605-9)

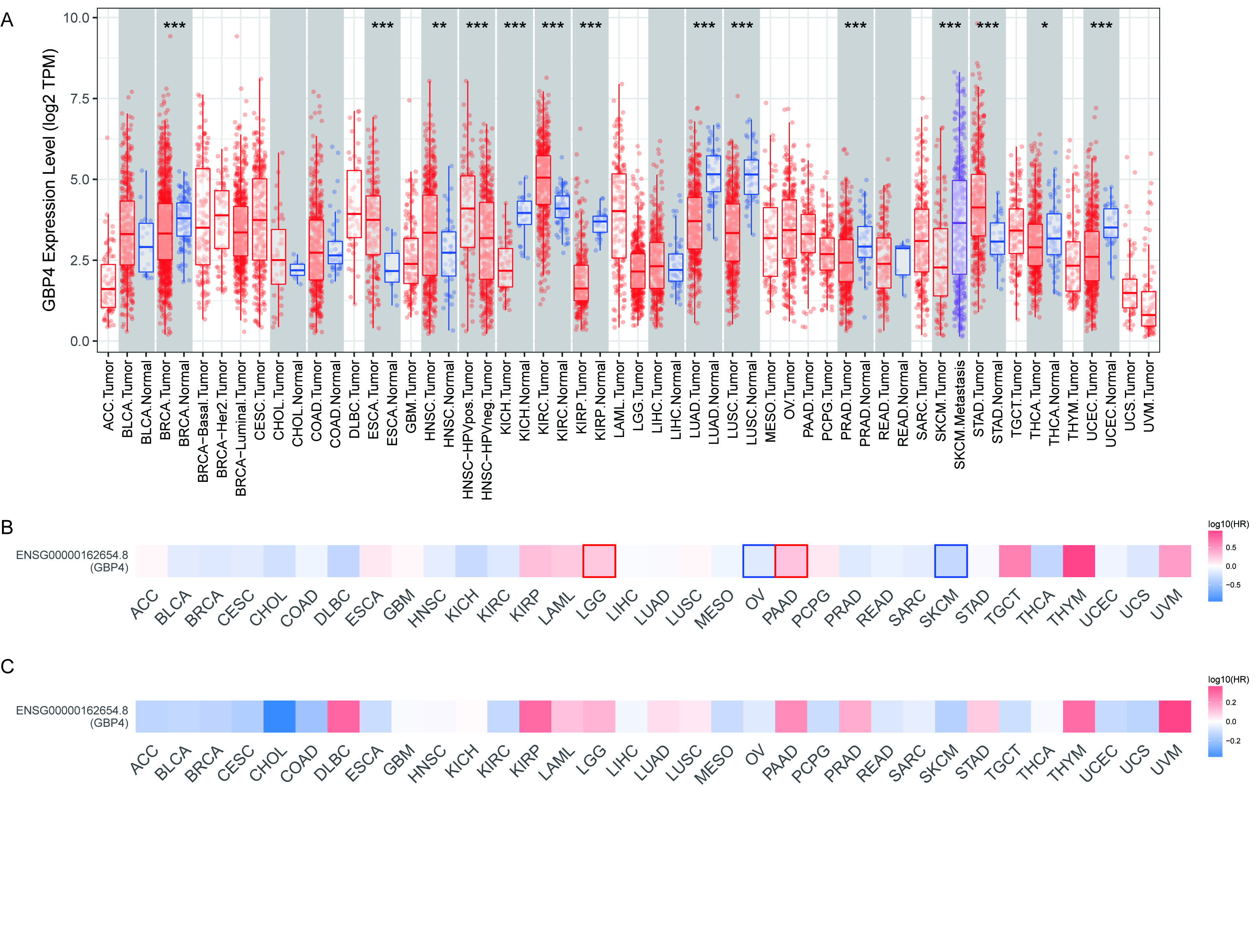

Supplement: Supplementary file 3 — Supplementary file3 (TIF 6065 KB) Supplementary Figure 1.Pan-cancer analysis of expression and prognostic value of GBP4. (A)Expression of IFITM3 across different cancer types. Heatmap of prognostic value of GBP4 in predicting (B) OS and (C) PFS in pan-cancer analysis. Expression of STAT1 is positively correlated with GBP4 at mRNA level (D) and protein level (E) [file 432_2024_5605_MOESM3_ESM.tif]

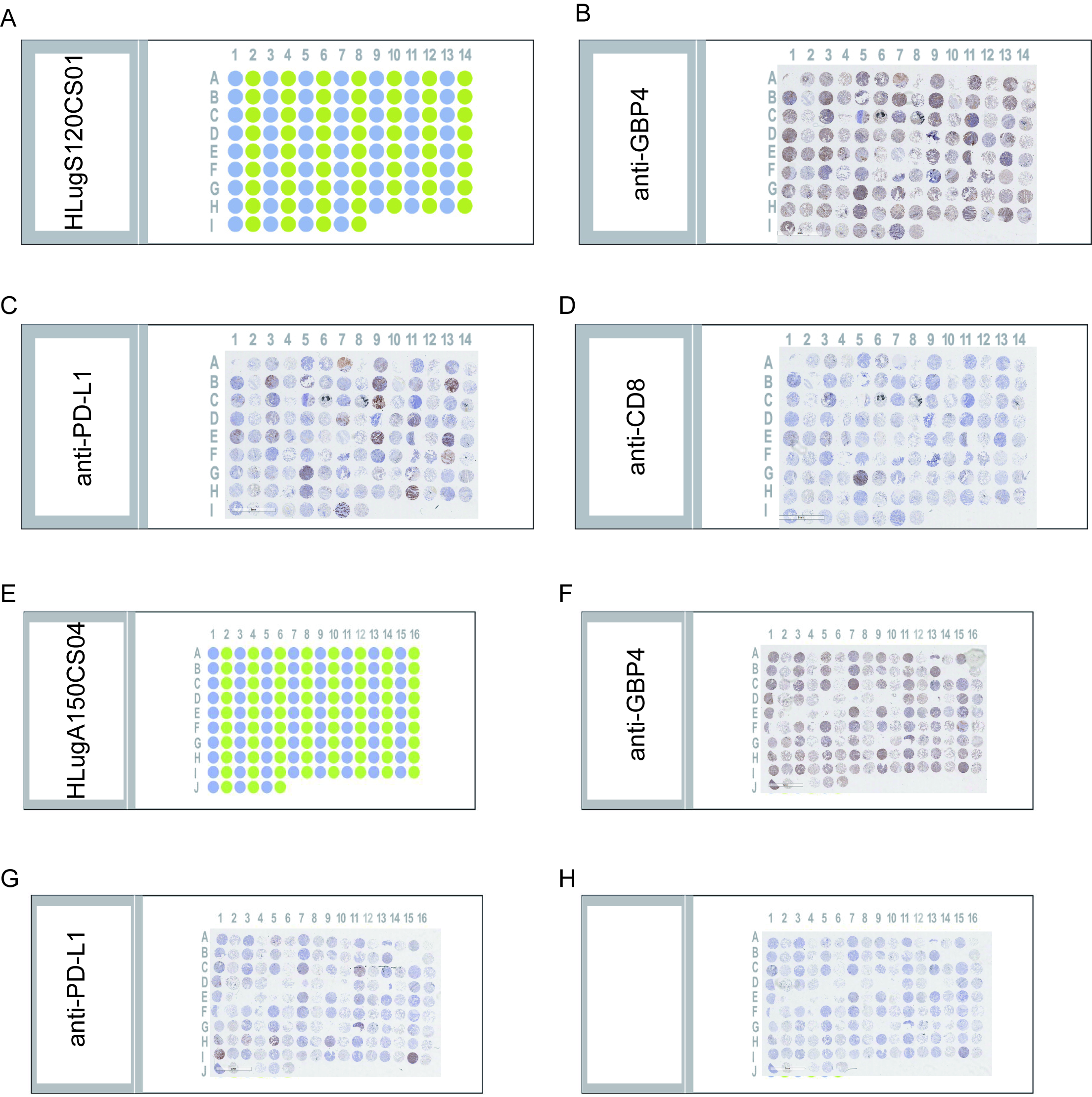

Supplement: Supplementary file 4 — Supplementary file4 (TIF 8718 KB) Supplementary Figure 2. The landscape of TMA HLugS120CS01 and HLugA150CS04. (A) Distribution of samples in TMA HLugS120CS01. Blue dots: tumor samples; Green dots: para-tumor samples. (B) The landscape of anti-GBP4 staining TMA HLugS120CS01. (C) The landscape of anti-PD-L1 staining TMA HLugS120CS01. (D) The landscape of anti-CD8 staining TMA HLugS120CS01. (E) Distribution of samples in TMA HLugA150CS04. Blue dots: tumor samples; Green dots: para-tumor samples. (F) The landscape of anti-GBP4 staining TMA HLugA150CS04. (G) The landscape of anti-PD-L1 staining TMA HLugA150CS04. (H) The landscape of anti-CD8 staining TMA HLugA150CS04 [file 432_2024_5605_MOESM4_ESM.tif]

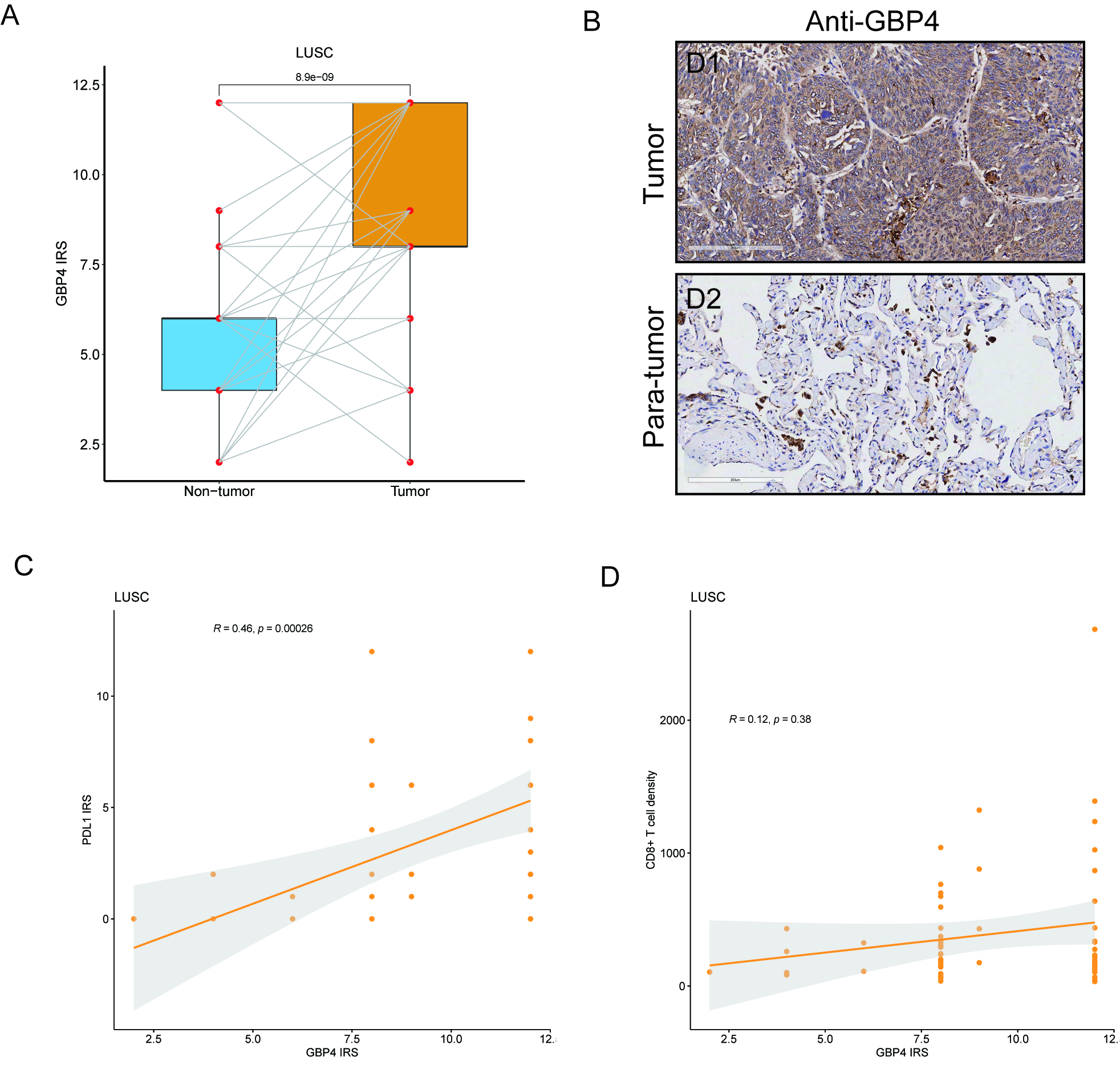

Supplement: Supplementary file 5 — Supplementary file5 (TIF 5324 KB) Supplementary Figure 3. The role of GBP4 in predicting clinical and immune phenotypes in the recruited TMA cohort. (A) Expression levels of GBP4 in tumor and paratumor tissues. (B) Representative images revealing GBP4 expression in tumor and paratumor tissues using anti-GBP4 staining. Magnification, 200X [file 432_2024_5605_MOESM5_ESM.tif]

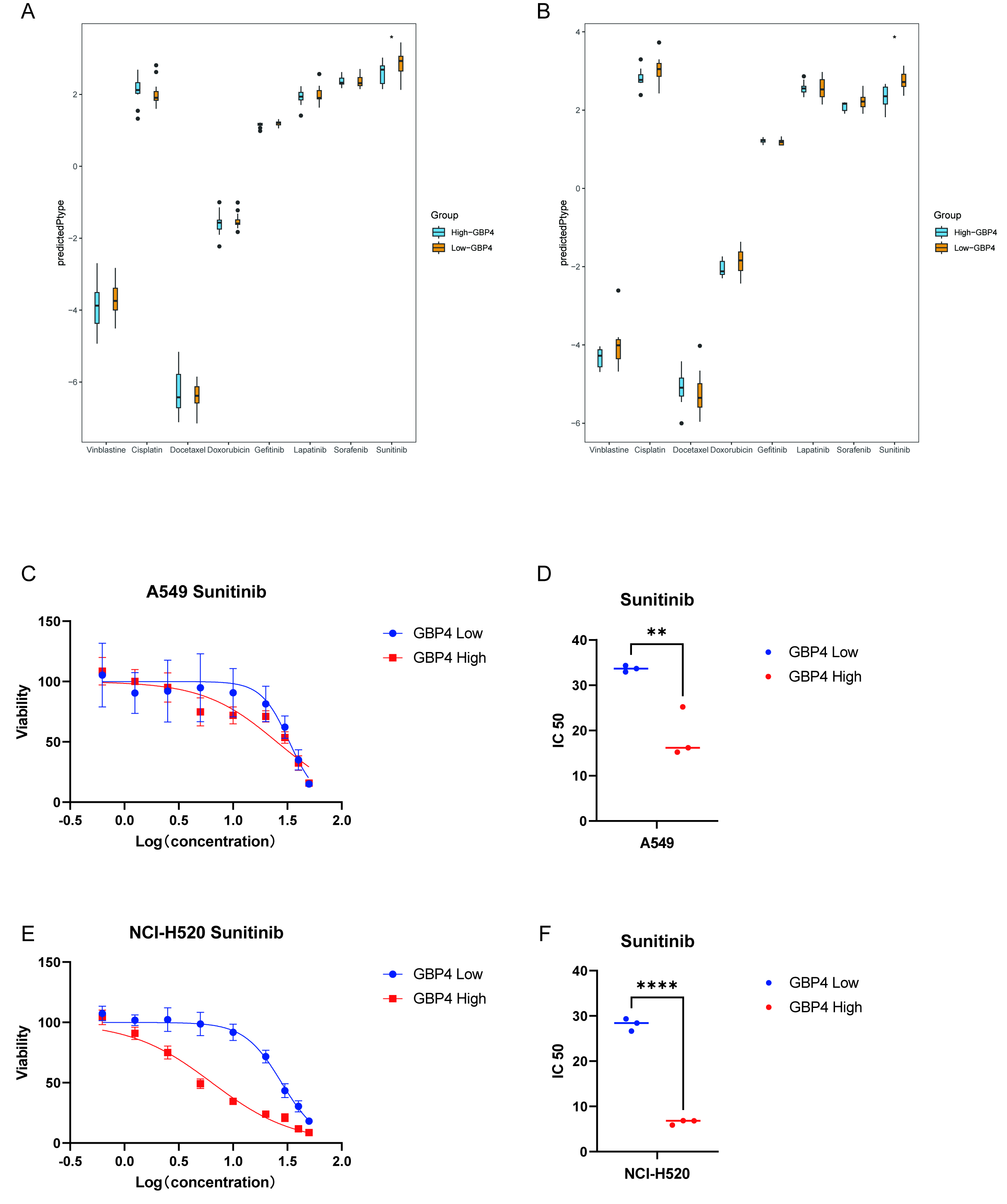

Supplement: Supplementary file 6 — Supplementary file6 (TIF 26989 KB) Supplementary Figure 4. GBP4 Can be used to predict therapeutic opportunities in NSCLC. Differences in the IC50s of common anticancer drugs calculated using the GSE datasets GSE135222 (A) and GSE126044 (B). (C,D) Differences in the IC50s of sunitinib in A549 cell line. (E,F) Differences in the IC50s of sunitinib in NCI-H520 cell line [file 432_2024_5605_MOESM6_ESM.tif]
